# VAMP7 Modulates Ciliary Biogenesis in Kidney Cells

**DOI:** 10.1371/journal.pone.0086425

**Published:** 2014-01-22

**Authors:** Christina M. Szalinski, Anatália Labilloy, Jennifer R. Bruns, Ora A. Weisz

**Affiliations:** 1 Renal Electrolyte Division, University of Pittsburgh Medical School, Pittsburgh, Pennsylvania, United States of America; 2 Department of Cell Biology, University of Pittsburgh Medical School, Pittsburgh, Pennsylvania, United States of America; 3 Ciência sem Fronteiras, CNPq, Brasilia, Brazil; Institut Jacque Monod, Centre National de la Recherche Scientifique, France

## Abstract

Epithelial cells elaborate specialized domains that have distinct protein and lipid compositions, including the apical and basolateral surfaces and primary cilia. Maintaining the identity of these domains is required for proper cell function, and requires the efficient and selective SNARE-mediated fusion of vesicles containing newly synthesized and recycling proteins with the proper target membrane. Multiple pathways exist to deliver newly synthesized proteins to the apical surface of kidney cells, and the post-Golgi SNAREs, or VAMPs, involved in these distinct pathways have not been identified. VAMP7 has been implicated in apical protein delivery in other cell types, and we hypothesized that this SNARE would have differential effects on the trafficking of apical proteins known to take distinct routes to the apical surface in kidney cells. VAMP7 expressed in polarized Madin Darby canine kidney cells colocalized primarily with LAMP2-positive compartments, and siRNA-mediated knockdown modulated lysosome size, consistent with the known function of VAMP7 in lysosomal delivery. Surprisingly, VAMP7 knockdown had no effect on apical delivery of numerous cargoes tested, but did decrease the length and frequency of primary cilia. Additionally, VAMP7 knockdown disrupted cystogenesis in cells grown in a three-dimensional basement membrane matrix. The effects of VAMP7 depletion on ciliogenesis and cystogenesis are not directly linked to the disruption of lysosomal function, as cilia lengths and cyst morphology were unaffected in an MDCK lysosomal storage disorder model. Together, our data suggest that VAMP7 plays an essential role in ciliogenesis and lumen formation. To our knowledge, this is the first study implicating an R-SNARE in ciliogenesis and cystogenesis.

## Introduction

The directional transfer of membrane and soluble proteins from one cellular compartment to another is essential for cell survival. A critical step in these membrane trafficking events is the selective fusion of vesicles with target organelles. SNAREs (Soluble *N*-ethylmaleimide-sensitive factor Attachment protein REceptors) are key components of the machinery required to maintain selectivity, and are directly responsible for fusion. These small proteins localize to organelle and vesicle membranes and interact when the two membranes are in close proximity. The energy released from their interaction is thought to drive fusion [1].

One R-SNARE on the vesicle membrane and three Q-SNAREs on the target membrane interact to form a helical bundle. In general, four distinct SNAREs participate in the helical bundle, although there are some Q-SNAREs (members of the SNAP25 subfamily) that contribute two helices to the coil [2]. Unlike most SNAREs, which are transmembrane proteins, these Q-SNAREs that contain two SNARE motifs are palmitoylated [1]. A subset of R-SNAREs fall into a family known as VAMPs (Vesicle Associated Membrane Proteins), based on their initial discovery in synaptic vesicle membranes.

Newly synthesized proteins must be properly transported to the apical or basolateral domain to maintain epithelial cell function. A number of SNAREs have been implicated in polarized trafficking. Although their localizations were initially controversial, it has since been well established that the Q-SNAREs syntaxin 3 and syntaxin-4 localize to the apical and basolateral surfaces, respectively, in the kidney and in MDCK cells [3]–[6]. The R-SNARE VAMP3 (aka cellubrevin) has been suggested to pair with syntaxin-4 in basolateral delivery [7], however, the R-SNAREs required for apical trafficking pathways have not been identified. Some studies have suggested that VAMP7 and VAMP8 are involved in apical trafficking [8], [9] but no defects in cell polarity have been observed in knockout mice [10], [11].

Many unique transport pathways exist for newly synthesized proteins to reach the apical and basolateral domains of polarized cells [12]. Newly synthesized proteins are directed towards particular delivery routes depending on their sorting determinants [13], [14]. In many cases, post-Golgi trafficking of these proteins involves intermediate transport through endocytic compartments prior to surface delivery. Many apical proteins traffic through the Rab11-positive Apical Recycling Endosome (ARE) prior to arrival at the apical surface, and there appear to be multiple, differentially regulated exit pathways from this compartment [14], [15]. Endolyn, a transmembrane protein sorted by an N-glycan dependent mechanism, traffics through the ARE *en route* to the apical surface. From this compartment, endolyn is delivered to the apical membrane via a pathway that requires the motor protein myosin Vb [13]. In contrast, a truncated, soluble version of endolyn (Ensol), traverses the ARE but its apical secretion is independent of myosin Vb activity [15]. Other apical proteins, including the lipid-raft associated protein influenza hemagglutinin (HA), appear to bypass the ARE and may instead transit apical early endosomes [13]. The VAMPs that mediate fusion of these distinct endosome-derived vesicles with the apical surface have not been identified.

Recent studies in other epithelial cell types have implicated a role for VAMP7 in a subset of apical delivery events. In polarized Fischer rat thyroid cells, where apically destined proteins are vectorially delivered to the cell surface, knockdown of VAMP7 disrupted apical delivery of both the lipid-raft associated protein placental alkaline phosphatase (PLAP) and the lipid-raft independent protein dipeptidylpeptidase IV (DPPIV) [9]. Different results were obtained in the intestinal epithelial cell line Caco-2, which use both transcytotic and vectorial routes to deliver newly synthesized proteins to the apical surface [16], [17]. In these cells, delivery of DPPIV, which traffics primarily through the transcytotic pathway, was unaffected by VAMP7 knockdown, whereas vectorial delivery of the lipid-raft dependent protein PLAP was disrupted [9].

Deciphering the role of VAMPs is complicated because SNAREs can assemble in many combinations to provide a large array of selective complexes. That said, there are redundancies in SNARE function, such that the same SNARE complex may function at multiple steps in membrane traffic. SNAP23 is involved in fusion of post-Golgi vesicles with the plasma membrane [18], [19], as well as in transcytosis [20]. Additionally, multiple SNARE complexes may mediate the same fusion pathway. For example, both VAMP7 and VAMP8 can form complexes with syntaxin-7 and both are involved in late-endosome to lysosome fusion [21]. Such redundancies have made it difficult to sort out the SNAREs involved in a given transport pathway.

In this study, we sought to investigate whether VAMP7 plays a role in any of the many delivery pathways to the apical surface of MDCK cells. VAMP7 is localized primarily in lysosomal compartments in many cell types, and has a well-established role in lysosomal delivery [21]–[31]. However, VAMP7 was also found to be enriched at the apical plasma membrane of polarized intestinal cells [8], and has been shown to complex with the apical Q-SNARE, syntaxin 3 [8], [18], [27]. Moreover, adding antibody against VAMP7 to permeabilized cells reduced the trans-Golgi network (TGN)- to-apical surface transport of HA in MDCK cells [18]. Surprisingly, however, we found that siRNA-mediated knockdown of VAMP7 had no effect on apical delivery of a variety of cargoes in MDCK cells. In contrast, we observed defects in ciliogenesis and in cystogenesis upon knockdown of VAMP7. To our knowledge, this is the first study implicating an R-SNARE in these cellular events.

## Results

### Expression and Subcellular Localization of VAMP Isoforms in MDCK Cells

**Figure 1:**
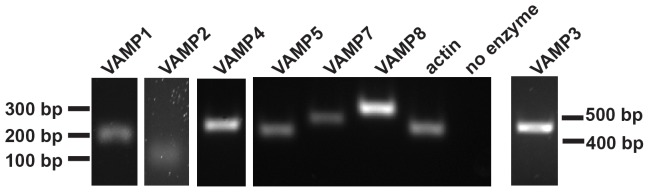
VAMP isoforms in MDCK cells. (A) mRNA isolated from MDCK cells and converted to cDNA was subjected to reverse transcription PCR using primers designed to detect VAMP isoforms. VAMP1 (218 bp expected product), VAMP2 (101 bp), VAMP3 (462 bp), VAMP4 (244 bp), VAMP5 (216 bp), VAMP7 (261 bp), and VAMP8 (298 bp) were detected. Actin (231 bp) was detected as an additional control to ensure amplification of mRNA and reverse transcriptase was excluded from the indicated sample as a negative control.

**Figure 2:**
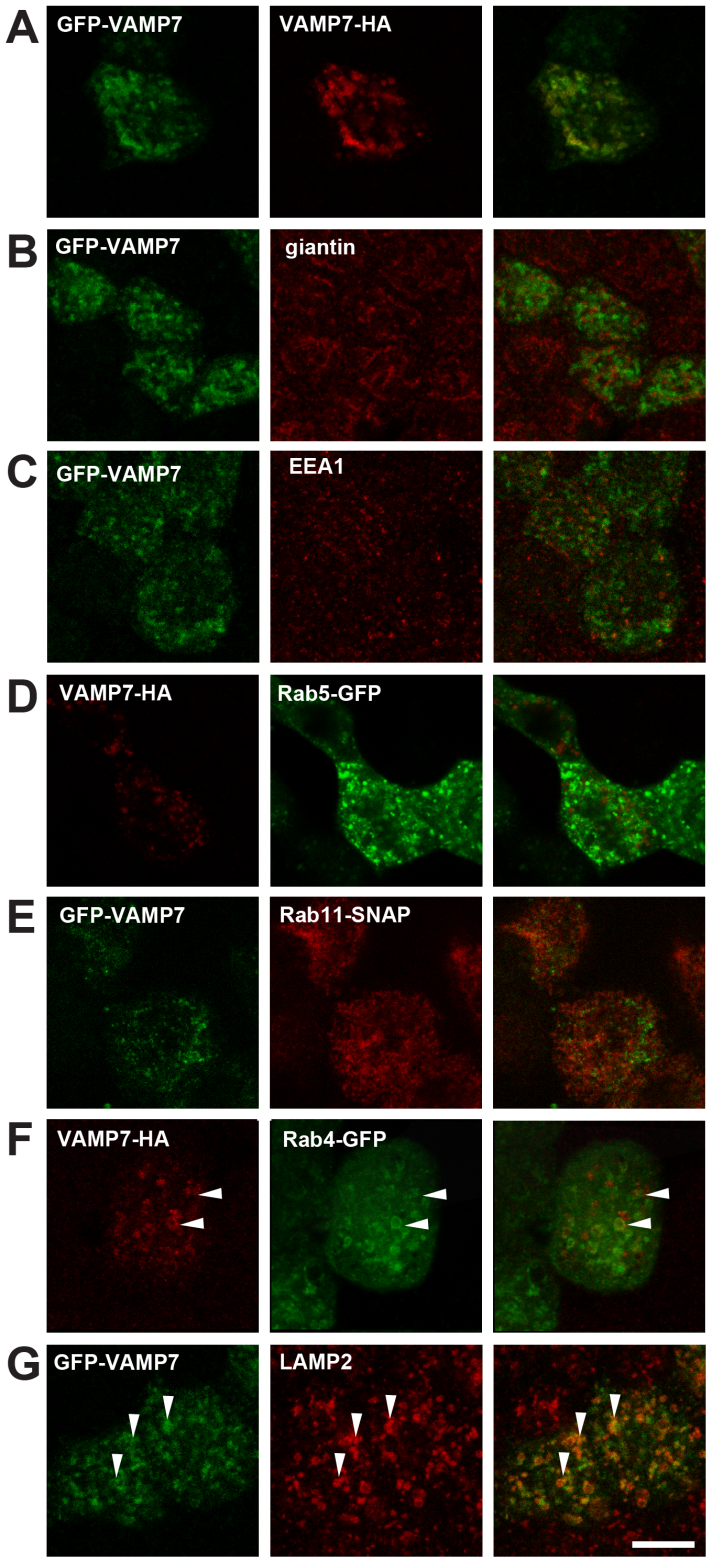
VAMP7 localization in polarized MDCK cells. Polarized MDCK cells grown on Transwells were transfected with GFP- or HA-tagged VAMP7 constructs and stained for the indicated endogenous proteins or co-transfected GFP- or SNAP-tagged organelle markers. (A) GFP-VAMP7 and VAMP7-HA colocalize with each other. Little colocalization is observed between VAMP7 and (B) giantin (Golgi), (C) EEA1 (early endosomes), (D) Rab5-GFP (early endosomes), or (E) Rab11a-SNAP. More extensive colocalization (marked by arrowheads) is observed between VAMP7 and (F) Rab4-GFP (recycling endosomes) and (G) LAMP2 (late endosomes and lysosomes).

Multiple VAMPs, including VAMP1, VAMP2, VAMP3, VAMP4, VAMP5, VAMP7 and VAMP8 are expressed in rat kidney [32]–[36]. All of these were readily detected by reverse transcription-polymerase chain reaction (RT-PCR) of RNA isolated from polarized MDCK cells (Figure 1). VAMP7 is associated with endosomes and lysosomes in many cell types, and we sought to confirm its localization in polarized MDCK cells. We were unable to detect endogenous VAMP7 in MDCK cells using commercially available antibodies, so we expressed low levels of tagged VAMP7 in these cells by transient transfection, then allowed the cells to differentiate on permeable supports prior to processing for immunofluorescence. Pearson’s correlation coefficients were determined using Imaris software for each of these markers (Table 1). VAMP7 proteins modified by a cytoplasmic amino-terminal GFP tag (GFP-VAMP7) or by a lumenally-oriented carboxy-terminal HA epitope tag (VAMP7-HA) colocalized with one another when coexpressed, suggesting that neither tag disrupts the targeting of heterologously expressed VAMP7 (Figure 2A; Table 1). Consistent with previous reports, we found little if any detectable VAMP7 at the plasma membrane at steady state (not shown and [30], [37]). GFP-VAMP7 colocalized poorly with the Golgi marker giantin (Figure 2B; Table 1) or with markers of early endosomes (EEA1, GFP-Rab5; Figure 2C and 2D; Table 1) or the ARE (SNAP-Rab11a; Figure 2E; Table 1) in polarized cells. More colocalization was observed with the endosome marker Rab4-GFP, which mediates fast recycling from early endosomes (Figure 2F; Table 1). The greatest extent of colocalization was observed with the lysosomal marker lysosome associated membrane protein 2 (LAMP2; Figure 2G; Table 1). We observed essentially similar results in subconfluent MDCK cells (data not shown). The observed colocalization between VAMP7 and LAMP2 is consistent with previous studies, which reported that VAMP7 colocalizes with lysosomal markers Niemann-Pick C1, lysosomal glycoprotein 120, LAMP1, and CD63 [22]–[25], [27], [28], [30], [31]. VAMP7 was also shown to partially colocalize with the transferrin receptor [another marker of recycling endosomes [22], [27]].

**Table 1:** Colocalization of VAMP7 with organelle markers.

	Pearson’s Correlation Coefficient
GFP-VAMP7 and VAMP7-HA	0.736±0.108
GFP-VAMP7 and giantin	0.170±0.060
GFP-VAMP7 and EEA1	0.151±0.055
Rab5-GFP and VAMP7-HA	0.379±0.188
Rab11a-SNAP and VAMP7-HA	0.351±0.222
Rab4-GFP and VAMP7-HA	0.504±0.099
GFP-VAMP7 and LAMP2	0.736±0.108

MDCK cells cultured on Transwell supports were transfected with VAMP7 constructs and where indicated, with tagged organelle markers or stained for endogenous organelle markers. Imaris software was used to determine the Pearson’s correlation coefficient between the markers. An R value of 1.0 would indicate perfect colocalization, however, variability in intensities of each channel can decrease the coefficient.

### VAMP7 Knockdown does not Affect Delivery of Apical Cargoes

**Figure 3:**
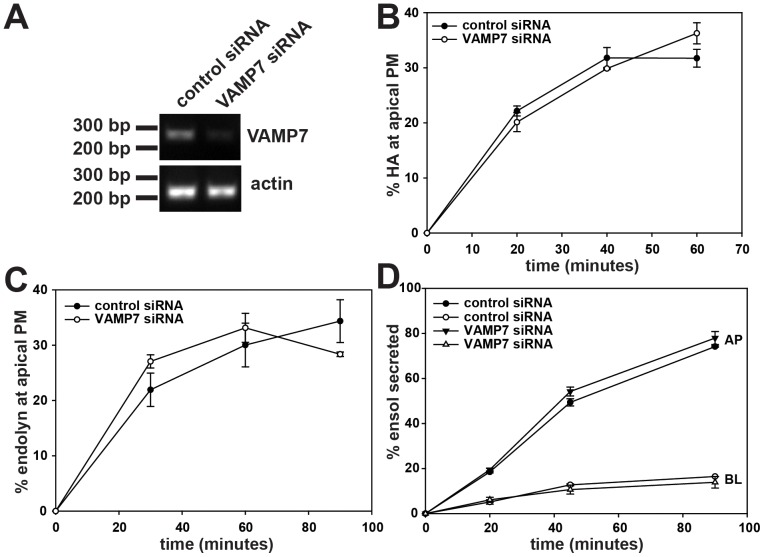
Knockdown of VAMP7 has no effect on apical secretion of lipid-raft associated, raft-independent, and secreted cargo. (A) MDCK cells were transfected with control siRNA or siRNA targeting VAMP7, and knockdown after four days was assessed using RT-PCR. A representative gel is shown. We routinely observed approximately 80% knockdown using this oligonucleotide. (B) Apical delivery kinetics of the lipid raft associated protein HA in MDCK cells transfected with control or VAMP7 siRNA were quantitated using a cell surface trypsinization assay as described in Methods. Results from a representative experiment performed in duplicate are plotted (mean +/− range). Similar results were obtained in three experiments. (C) Apical delivery kinetics of the raft-independent protein endolyn were quantitated using a surface biotinylation assay as described in Methods. The results are representative (mean +/− range of duplicate samples) of two independent experiments. (D) Apical (AP) and basolateral (BL) secretion kinetics of Ensol were measured in control and VAMP7 knockdown cells as described in Methods. A representative experiment (mean +/− range of duplicate samples) is shown. Similar results were obtained in five experiments.

Multiple pathways exist for newly synthesized proteins to reach the apical membrane of polarized cells [12]. Endolyn traverses the Rab11-positive ARE *en route* to the apical surface, and is delivered to the apical surface via a pathway that requires the motor protein myosin Vb [13]. In contrast, the lipid-raft-associated protein influenza HA is apparently excluded from the ARE [13]. Soluble proteins also apparently enter the ARE but can exit via myosin Vb-dependent or independent pathways [15]. Based on previous observations implicating VAMP7 in apical biosynthetic delivery [8], [9], we hypothesized that VAMP7 may play a role in apical delivery of a subset of apical cargo in MDCK cells [15]. To test our hypothesis we knocked down VAMP7 in MDCK cells and determined using biochemical approaches whether apical delivery of various membrane and secreted proteins known to take distinct routes to the surface is compromised. We tested the efficacy of multiple siRNAs targeting canine VAMP7 using RT-PCR to estimate the extent of knockdown. Of these, we selected a siRNA that consistently achieved ∼80% knockdown of VAMP7 mRNA (Figure 3A). The efficiency of knockdown was confirmed by qPCR (Fig. S1). Importantly, knockdown of VAMP7 did not reduce the levels of other VAMPs expressed in MDCK cells as assessed by quantitative PCR (qPCR) (Fig. S1). Cells transfected with control or VAMP7 siRNA were plated on permeable supports for four days prior to quantitating the effect of knockdown on the kinetics of surface delivery using a pulse chase approach as described in Materials and Methods. Knockdown of VAMP7 had no effect on apical delivery of the lipid-raft associated protein influenza HA, which bypasses the ARE *en route* to the surface [Figure 3B, [13]], Similarly, we observed no effect of VAMP7 knockdown on apical delivery kinetics of the glycan-dependent protein endolyn, which traffics through the Rab11-positive ARE and is delivered to the apical surface in a myosin Vb-dependent manner [Figure 3C, [13]]. Additionally, we observed no effect on the apical secretion of Ensol, a truncated version of endolyn that transits the ARE but unlike endolyn, does not require Myosin Vb for apical delivery [Figure 3D, [15]]. VAMP7 knockdown also had no effect on the kinetics or polarity of delivery of two other apically-destined proteins [the multiligand receptor megalin and secreted glycosylated growth hormone [15], [38]; data not shown].

### VAMP7 Knockdown Decreases Cilia Length and Frequency

**Figure 4:**
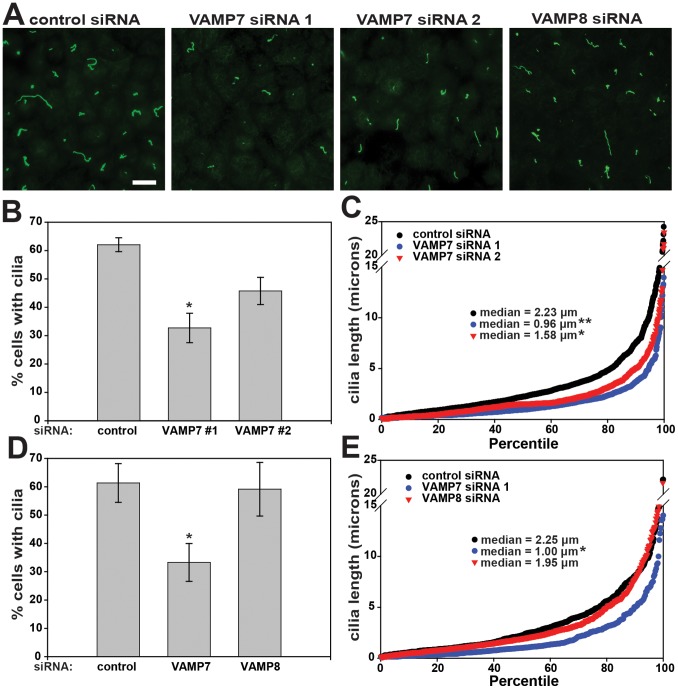
VAMP7 knockdown decreases cilia frequency and cilia length in MDCK cells. (A) MDCK 2001 cells were transfected as indicated with control siRNA, our standard VAMP7 siRNA (VAMP7 #1), a less effective VAMP7 targeting sequence (VAMP7 #2), or VAMP8 siRNA. Cells were cultured for four days on permeable supports, then fixed and processed for indirect immunofluorescence to detect acetylated tubulin and cell nuclei. Representative fields acquired using epifluorescence microscopy are shown. Scale bar = 10 µm. (B, D) The number of cells in multiple fields was determined using DAPI nuclear stain and used to quantify the fraction of cells with a primary cilium in each sample. The average of three experiments is plotted (mean +/− SEM); *p<0.05 compared to control using one-way ANOVA with Bonferroni correction. (B) Control n = 1,907; VAMP7 n = 1,747; VAMP7 #2 n = 1,608 cells. (D) Control n = 1,040, VAMP7 n = 1,169, VAMP8 n = 1,049 cells. (C, E) Cilia lengths in multiple fields were measured using ImageJ software, then sorted by length in ascending order and graphed by percentile (100^th^ percentile = longest cilium in each sample). Each point on the graph represents the length of an individual cilium. (C) There was a statistically significant reduction in cilia length in cells transfected with VAMP7 #1 and VAMP7 #2 siRNAs, as assessed by comparing the medians from four experiments (*p<0.05, **p<0.01 using one-way ANOVA with Bonferroni correction). (E) There was a statistically significant reduction in cilia length in VAMP7 depleted cells, as assessed by comparing the medians from three experiments (*p<0.05 using one-way ANOVA with Bonferroni correction).

The primary cilia of polarized kidney cells are specialized apical structures believed to function as mechanosensors that sense changes in flow to modulate downstream signaling pathways [39]. Although little is known about how proteins traffic to this compartment, some ciliary proteins are thought to be delivered to the apical plasma membrane prior to reaching the primary cilium [40]. Transport to the ciliary membrane may then occur by lateral diffusion across a septin barrier [41], [42]. Because one of the VAMP7 cognate SNAREs, syntaxin 3, was found to be involved in ciliogenesis [43] we asked whether VAMP7 also has a role in this process. To this end, we transfected MDCK cells with control or VAMP7 siRNA, plated the cells on permeable supports, and processed them after four days for indirect immunofluorescence to detect the cilia marker acetylated tubulin [44]. Images of randomly selected fields were acquired using an epifluorescence microscope, and cilia length and the percent of cells elaborating a primary cilium were quantified in randomly selected fields using ImageJ. As shown in Figure 4 (panels A, C, E), we found a statistically significant decrease in the median length of cilia in cells depleted of VAMP7 compared with control cells (Figure 4 C,E). Additionally, the fraction of cells expressing a primary cilium was significantly decreased (Figure 4 B,D). Similar results were obtained in the rat neuronal cell line PC-12 (Figure S2). In contrast, knockdown of VAMP8 had no effect on either cilia length or frequency (Figure 4A, 4D).

Several known interacting partners for VAMP7 have previously been implicated in ciliogenesis. For example, MDCK cells depleted of the VAMP7-interacting partner syntaxin 3 had fewer and shorter cilia than control cells [43]. Moreover, knockdown of septin 7, a cytoskeleton-associated GTPase that localizes to the ciliary base and also binds to the VAMP7 partner AP-3, resulted in a decrease in cilia length and number [45], [46]. Thus, we asked whether depletion of VAMP7 would affect the steady state localization of syntaxin 3 and septin 7. Additionally, we tested whether the localization of Arl6/BBS3, a protein required for ciliary delivery of a subset of proteins is altered in cells treated with VAMP7 siRNA. As shown in Figure S3, we could find no obvious changes in the steady state distribution of any of these proteins upon knockdown of VAMP7.

Attempts to rescue the effect of VAMP7 knockdown on ciliogenesis by heterologous expression of human VAMP7 were unsuccessful. Indeed, we found that cells overexpressing VAMP7 also had somewhat shorter cilia compared with controls (not shown). Given that VAMP7 functions in a complex with other SNAREs, it is likely that its expression level relative to endogenous partners is critical for efficient function. Consistent with this idea, overexpression of syntaxin 3 has been demonstrated to disrupt the apical delivery of membrane and secreted proteins in Caco-2 cells [47]. As an alternative approach to confirm that the changes we observed were due to a specific consequence of VAMP7 depletion rather than to an off-target effect of our siRNA oligonucleotide, we determined the effect on ciliogenesis of a second siRNA oligonucleotide (VAMP7 #2) that targets a different region of VAMP7 and knocks down the protein with lower efficiency (∼50% by RT-PCR, data not shown). Transfection of MDCK cells with the VAMP7 siRNA #2 also led to a decrease in cilia length and frequency, but to a lesser extent than our primary siRNA, consistent with the reduced knockdown efficiency of this oligonucleotide (Figure 4B–D). Together, these data suggest that knockdown of VAMP7 causes a selective decrease in both cilia length and frequency.

Changes in cilia length have been linked to alterations in cell cycle and proliferation [48]. Thus, we asked whether the effect of VAMP7 depletion on ciliogenesis could be due to changes in cell proliferation [49]. Indeed, overexpression of the cytosolic fragment of VAMP7 has been shown to inhibit cytokinesis in BSC1 cells, resulting in multiploidy [50]. Our studies were performed using differentiated, superconfluent MDCK cells, the majority of which should be quiescent. To ensure quiescence, we serum starved MDCK cells transfected with control or VAMP7 siRNA for 48 h prior to quantifying cilia length and frequency. Similar to our normal conditions, knockdown of VAMP7 in starved cells resulted in a decrease in both of these parameters (data not shown). Additionally, to confirm that VAMP7 does not alter proliferation in our system, we quantitated the number of nuclei per field in control and VAMP7 knockdown cells by DAPI staining. In six experiments, we observed no significant difference (control average = 183+/−14 cells/field, VAMP7 KD average = 186+/−6 cells/field). Together, these data suggest that the effect of VAMP7 knockdown on ciliogenesis is not due to effects on the cell cycle.

### VAMP7 Expression Modulates Lysosome Size

**Figure 5:**
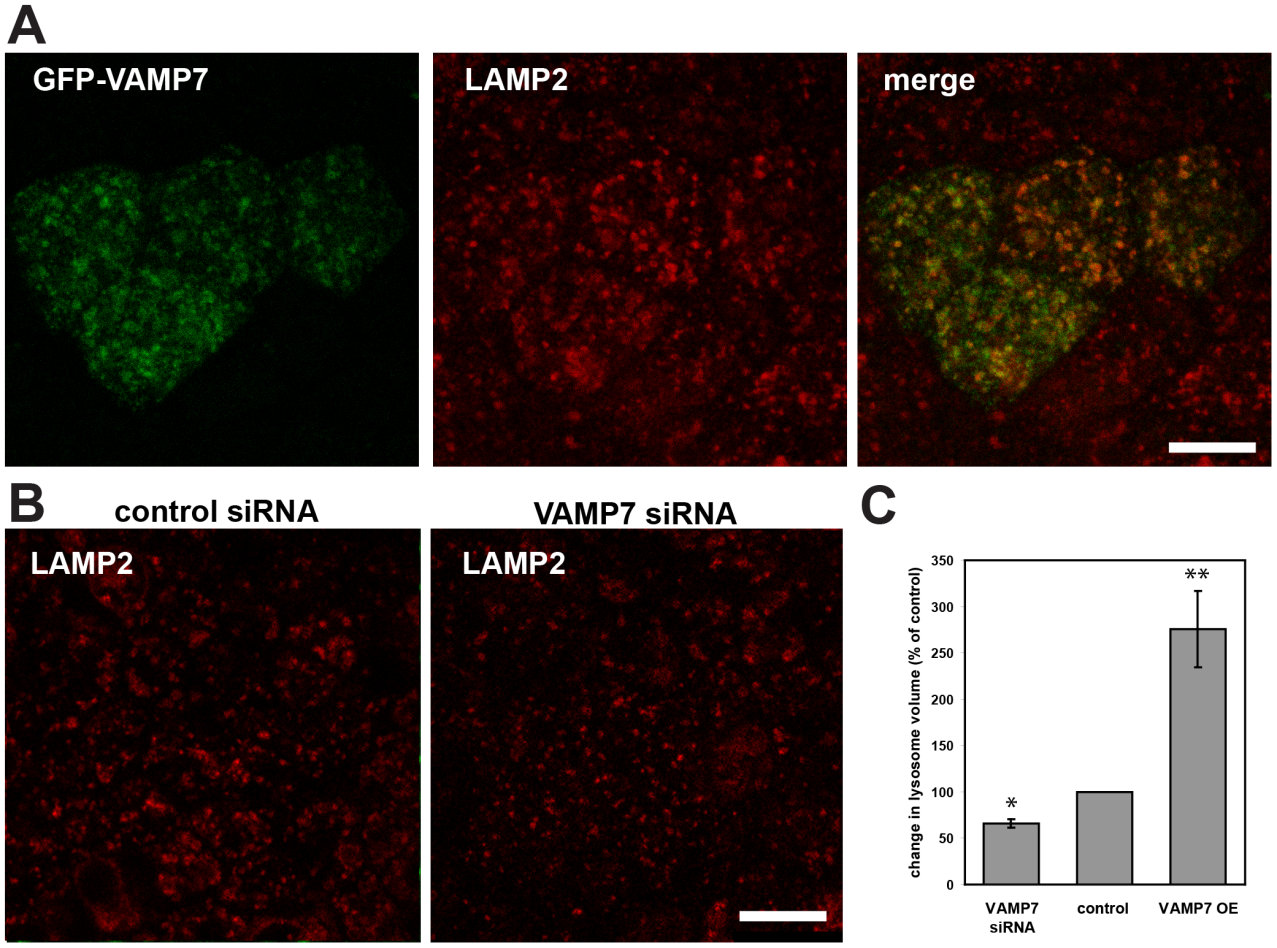
VAMP7 knockdown and overexpression alters lysosome volume. MDCK cells were transfected with cDNA encoding GFP-VAMP7 (A) or with control or VAMP7 siRNA (B) and processed for immunofluorescence to detect LAMP2 after four days. (C) Percent change +/− standard error of the mean in lysosome volume normalized to its respective control was quantitated using Imaris software as described in Methods. Compared to cells transfected with control siRNA, cells treated with VAMP7 siRNA had smaller LAMP2-positive structures compared to control. In contrast, VAMP7 overexpression (OE) led to an enlargement of LAMP2-positive structures. A total of ten stacks in two experiments were quantitated Scale bars = 10 µm; (* p<0.01 using two-sample t-test; **p<0.001 using paired t-test; see Methods for details.).

VAMP7 has previously been shown to play a role in the delivery of cargo from endosomes to lysosomes. Inhibition of VAMP7 function decreased EGF degradation in HeLa and reduced delivery of internalized dextran and BSA to lysosomes in MDCK cells and NRK cells, respectively [21], [23], [29]. To test whether VAMP7 is important for lysosome biogenesis in MDCK cells, we modulated VAMP7 levels by overexpression of GFP-VAMP7 or by siRNA-mediated knockdown and examined the consequences on lysosome size. Overexpression of VAMP7 led to enlargement of LAMP2-positive organelles (Figure 5A, 5C) and reduced their number by 68 percent (data not shown). In contrast, knockdown of VAMP7 caused a reduction in the average volume of LAMP2-positive structures (Figure 5A, 5C), but increased the number of LAMP2 structures by 138 percent (data not shown). These data suggest that, similar to its role in other cell types, VAMP7 is involved in delivery to lysosomes in MDCK cells.

### Perturbation of Lysosome Function *per se* has no Effect on Cilia Length

**Figure 6:**
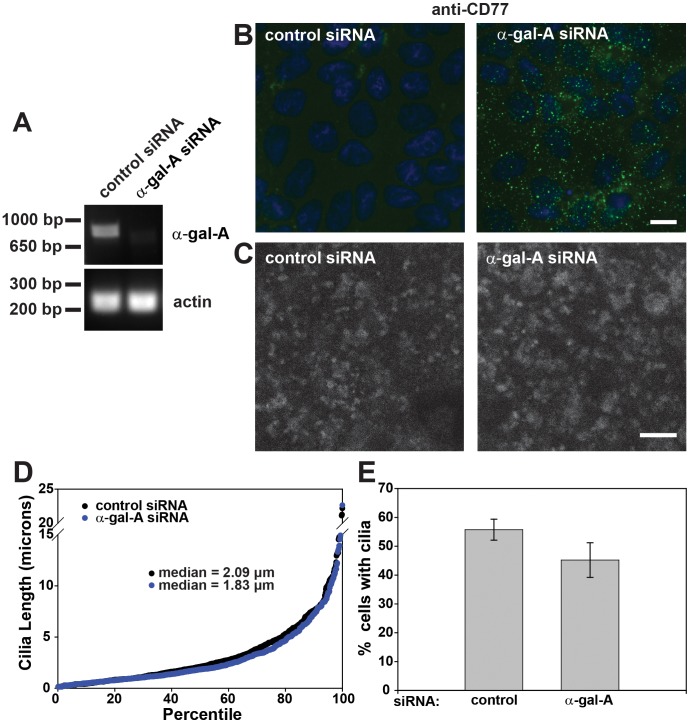
Perturbing lysosomes has no effect on ciliogenesis. MDCK cells were transfected with control or α-gal-A siRNA and cultured for four days on permeable supports, then harvested for (A) RT-PCR (expected products are 898 bp for α-gal-A and 231 bp for actin) or (B–E) processed for immunofluorescence. (B) Knockdown of α-gal-A caused a dramatic cellular accumulation of its substrate Gb3, as shown by indirect immunofluorescence using the anti-Gb3 antibody CD77. Scale bar = 10 µm. (C) Knockdown of α-gal-A lead to an increase in late-endosome/lysosome size, and a decrease in the number of these structures, shown by indirect immunofluorescence of LAMP2. Scale bar = 5 µm. (D) Cilia were detected using anti-acetylated tubulin antibody, and cilia from random fields were measured and counted using ImageJ. The cilia were sorted by length in ascending order and graphed by percentile as in Fig. 4. (E) The number of cilia in each field was divided by the number of nuclei (identified using DAPI staining) and the mean +/− SEM of three experiments is shown. There is not a significant difference in cilia length or in the percent of cells with cilia in control and α-gal-A siRNA transfected cells as assessed by t-test of the medians in three experiments. Data represent measurements from 1,535 cells for control siRNA and 1,407 cells for α -gal-A siRNA-treated cells. Scale bar = 10 µm.

We considered the possibility that the consequences of VAMP7 knockdown on lysosomal size/function and on ciliogenesis could be directly linked. In support of the latter possibility, siRNA mediated depletion of a protein implicated in the delivery of EGF to lysosomes (PTPN23) was also shown to result in fewer cilia in retinal epithelial cells [46], [51]. To test whether there is a connection between lysosome dysfunction and aberrant ciliogenesis, we examined cilia length in an MDCK model for lysosomal storage disease. Fabry disease is caused by mutations in the lysosomal enzyme α-gal A that prevents normal catabolism of the glycolipid globotriaosylceramide [Gb3; [52]]. Transfection of MDCK cells with an siRNA targeting α-gal A resulted in efficient knockdown of mRNA as assessed by qPCR (89% reduction; Figure 6A) as well as in the dramatic increase in accumulation of Gb3 (measured using the anti-Gb3 antibody CD77; Figure 6B). VAMP7 levels in α-gal A knockdown cells were not different from control, as confirmed by qPCR (1.34 fold change; data not shown). α-gal A knockdown led to a 167% increase in lysosome volume (Figure 6C; data not shown). Neither cilia length (Figure 6D) nor frequency (Figure 6E) was significantly altered in α-gal A-depleted cells compared with controls, suggesting that there is no direct link between disrupted lysosome function and ciliogenesis.

### VAMP7 Knockdown Leads to Aberrant Cyst Morphology

**Figure 7:**
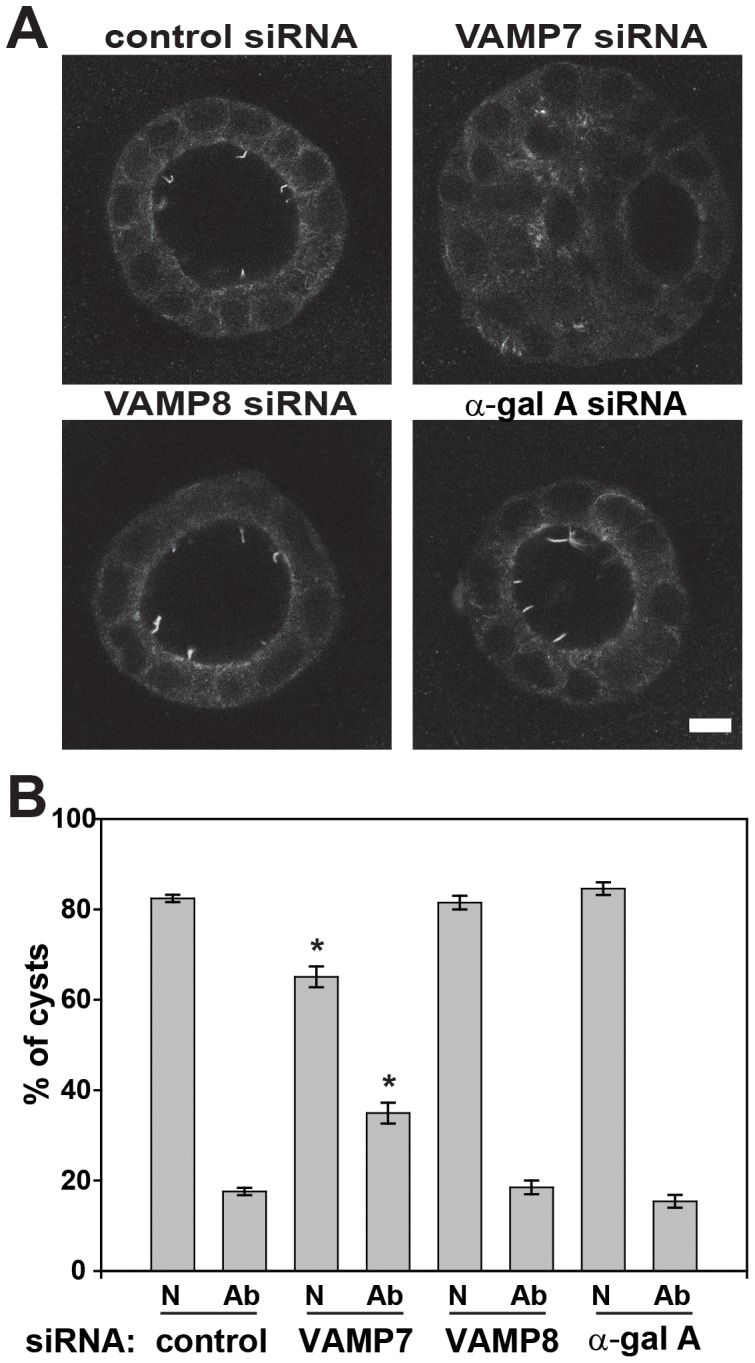
VAMP7 knockdown disrupts cyst morphology. MDCK cells were transfected with control, VAMP7, VAMP8 or α-gal A siRNA as indicated, then trypsinized and resuspended in basement membrane matrix the following day. Six days later, the cells were fixed and processed for immunofluorescence with anti-acetylated tubulin antibody to detect cilia. Confocal imaging revealed normal hollow lumens and cells with cilia projecting into the lumen in control, VAMP8 or α-gal A siRNA treated cells. However, transfection with VAMP7 siRNA led to an increase in the fraction of abnormal cysts with either filled lumens or multiple lumens. (A) Representative examples of a normal cyst in control siRNA-treated cells and an abnormal cyst in a VAMP7 siRNA treated sample are shown. (B) One hundred cysts from each siRNA treatment were classified as normal (N) or abnormal (Ab) in three experiments. *p<0.001 using one-way ANOVA with Bonferroni correction.

Many proteins that have been implicated in ciliogenesis also have a functional effect on cyst formation [43], [44], [53]–[57]. Thus, we tested whether knockdown of VAMP7 affects the formation of hollow cysts when MDCK cells are grown in a three-dimensional basement membrane Matrigel matrix for six days. Cells transfected with control siRNA predominantly formed cysts with a single hollow lumen (Figure 7A, 7B). In contrast, knockdown of VAMP7 led to a significant decrease in the number of normal lumens and an increase in the number of cysts with abnormal cysts with either no lumen or with multiple lumens (Figure 7A, 7B), though the majority of abnormal cysts had filled lumens. Knockdown of another R-SNARE, VAMP8, had no effect on cystogenesis, indicating that the effect is specific to VAMP7 (Figure 7A, 7B). Additionally, α-gal A knockdown showed no change in cystogenesis indicating that the effect of VAMP7 on cystogenesis is independent of its effects on lysosome fusion (Fig. 7A, 7B). This suggests that the effect of VAMP7 knockdown on cilia length has functional consequences on lumen formation.

## Discussion

Individual SNARE proteins localize to distinct plasma membrane subdomains of polarized epithelial cells to provide specificity in vesicle fusion with these domains. Here, we investigated the role of VAMP7 in lysosomal and apical delivery in MDCK cells. We found that VAMP7 is predominantly localized to late-endosomes and lysosomes, and is partially associated with recycling endosomes. Both VAMP7 knockdown and overexpression altered the size and number of LAMP2-positive compartments, consistent with previous reports demonstrating a role for this SNARE protein in late-endosome-to-lysosome fusion. Though VAMP7 has been previously implicated in apical trafficking in several epithelial cell types, we observed no effect of VAMP7 knockdown on the delivery of a number of cargoes known to take distinct routes to the apical surface in MDCK cells. However, we did observe a significant reduction in the fraction of cells expressing a primary cilium as well as a decrease in mean cilia length upon VAMP7 knockdown. Our results suggest that VAMP7 plays an important role in delivery to lysosomes and ciliogenesis in MDCK cells, but is not essential for surface delivery of apical proteins.

VAMP7 is unique among VAMPs in that it has an N-terminal extension of 120 amino acids termed the longin domain. There are two additional R-SNAREs with longin domains: Ykt6, and Sec22 [58]. Intriguingly, these three longin domain-containing VAMPs are the only R-SNAREs that are conserved in all eukaryotes, suggesting that the longin domain may have essential functions in trafficking [58]. Consistent with previous reports [21]–[31], we found that VAMP7 localizes predominantly to LAMP2-positive structures that include late endosomes and lysosomes. VAMP7 localization to these compartments is mediated by the interaction of its longin domain with the adaptor protein AP-3 [27], [29], [59]. AP-3 has also been implicated in the surface delivery of VSV-G [60], and in LAMP1, LIMPII and CD63 delivery to lysosomes [61], [62]. Although VAMP7 has been previously implicated in apical trafficking [8], [9], [18] we observed no changes in the apical delivery of a number of proteins that take different routes to the surface. This is in contrast to published data in MDCK cells showing that adding antibody against VAMP7 to permeabilized cells reduced the TGN to apical surface transport of HA [18]. It is conceivable that sufficient VAMP7 remains after knockdown to enable apical delivery or that another VAMP with redundant function also operates in these pathways. Indeed, functional redundancies have made it difficult to sort out the SNAREs involved in a given transport pathway. For example, neither individual knockdown of VAMP1, VAMP2, VAMP3, VAMP4, VAMP5, VAMP7, or VAMP8, nor combined knockdown of VAMP3, VAMP4, VAMP7, and VAMP8 affected the exocytosis of growth hormone in neuronal C1 cells [63]. In other instances, the role for a given VAMP in a particular trafficking step became evident only when the need for transport was exacerbated. For example, constitutive mucin secretion is normal in the lung of the VAMP8 knockout mouse, whereas a defect in mucous secretion could be observed after stimulation with IL-13 [64]. It may be necessary to deplete multiple VAMPs or to somehow stress the secretory pathway upon VAMP7 knockdown in order to observe a defect in apical trafficking in MDCK cells.

The primary cilium is a specialized subdomain of the apical surface. Our data show that VAMP7 plays an important role in ciliogenesis; however, we do not yet know its role in this process. The cilium is too narrow to accommodate transport vesicles and diffusion of proteins into the cilium is restricted by a large protein complex at the transition zone [45], [65], [66]. Delivery of transmembrane proteins to the cilium is thought to happen in one of two ways. Some ciliary proteins are inserted first in the apical plasma membrane and then laterally cross the transition zone into the periciliary base, as has been observed for the Sonic hedgehog receptor, Smoothened [42]. Alternatively (or in addition), some proteins are delivered directly to the periciliary base [41]. From this region, membrane proteins are transported into the cilium by intraflagellar transport (IFT) mediated by IFT protein complexes that also contain motor proteins [49]. Although we did not detect any changes in the distribution of the BBSome-associated protein Arl6/BBS3 upon VAMP7 knockdown, more thorough studies will be necessary to determine whether VAMP7 facilitates the transport of ciliary proteins to the apical surface and/or directly to the periciliary base.

Although several known interacting partners for VAMP7 have also been implicated in ciliogenesis, our data suggest that the effects of VAMP7 knockdown are not due to disruption of their localization or function. Similar to VAMP7, loss of its interacting partner syntaxin 3 has been shown to disrupt ciliogenesis [43]. However, we observed no changes in syntaxin 3 localization upon VAMP7 knockdown. Another VAMP7 partner, SNAP25 (the neuronal-specific homolog of the ubiquitous SNAP23) localizes to cilia and has also been implicated in ciliogenesis [19], [67]. It is not known whether SNAP23 is involved in ciliogenesis. Additionally, AP-3, which binds to the longin domain of VAMP7, also interacts with Septin 7, a cytoskeleton-associated GTPase localized at the ciliary base [45], [46]. This interaction may localize VAMP7 to its site of function in ciliogenesis, in addition to its role in localizing VAMP7 to endosomes and lysosomes [27], [29], [59]. Septin 7 knockdown caused a decrease in cilia length and number [46], suggesting that this septin may function in ciliary delivery at the transition zone. We found no effect of VAMP7 knockdown on septin 7 distribution, suggesting that VAMP7 modulates ciliogenesis via an effect downstream of septin 7 interaction.

Our data suggest that there is not a direct connection between the effects of VAMP7 knockdown on lysosomal function and on the defects in ciliogenesis and cystogenesis that we observed. Knockdown of α-gal A, deficient in the lysosomal storage disorder Fabry disease, led to the cellular accumulation of Gb3 but caused no defect in ciliogenesis. This is consistent with data showing that the pharmacological inhibitor concanamycin A, which blocks lysosomal acidification, had no effect on cilia length [51]. However, knockdown of PTPN23, a protein tyrosine phosphatase implicated in delivery of EGF to lysosomes, was previously shown to decrease the fraction of cells elaborating a primary cilium [46], [51]. PTPN23 knockdown also led to decreased levels of transferrin receptor at the surface, likely due a recycling defect [51]. It is possible that the effect of PTPN23 knockdown on ciliogenesis is related to its function in endosomal sorting rather than trafficking to lysosomes [51]. This is an intriguing possibility considering that we found VAMP7 partially colocalized with Rab4-positive recycling endosomes, and a number of proteins involved in recycling have been implicated in ciliogenesis [46].

Together, our data suggest that protein delivery to cilia and lysosomes in MDCK cells is more heavily reliant on VAMP7 function compared with other pathways in which VAMP7 may be also involved. Our studies do not reveal the precise role of VAMP7 in ciliogenesis, but support the idea that its functions in ciliogenesis and cystogenesis are independent of its role in lysosomal fusion. Further studies will be required to determine which step(s) in ciliogenesis require VAMP7.

## Materials and Methods

### RT-PCR and qPCR

RT-PCR was performed as described in [68]. RNA was extracted using the Ambion RNAqueous phenol-free total RNA isolation kit. Turbo DNA-free™ kit (Ambion) was used to remove contaminating DNA from RNA preparation. RNA preparations were treated with Turbo DNase for 30 min at 37°C and DNAse inactivation reagent was added for 2 min at RT. Equal amounts of RNA (1–2 µg), 2 µl of Oligo(dT)Primer (Ambion) and water (nuclease-free, to a total volume of 12 µl) were mixed, heated at 72°C for 3 min, and placed on ice, then centrifuged briefly. Two µl of 10x RT buffer (Ambion), 2 µl 2.5 mM dNTP mix (Invitrogen), 0.5 µl Moloney Murine Leukemia Virus Reverse Transcriptase (MMLV-RT; Ambion) (or water for control samples), 0.5 µl of RNAse inhibitor (Ambion), and 2 µl nuclease-free water were added and the sample was incubated at 42°C for 1 h and then at 92°C for 10 min. A 3 µl aliquot of this reaction was mixed with 2.5 µl of 10 µM sense and antisense primers, 10 µl of 5x PCR buffer, 0.5 µl of enzyme (NEB Phusion), 3 µl of DMSO, and 31.5 µl of PCR grade water, placed into a 0.6-ml thin walled tube, and incubated in a Bio-Rad thermocycler. The cycle started at 95°C for 4 min followed by five cycles of PCR: 95°C for 30 sec, 65°C for 30 sec (decreased by 1°C each round), 72°C for 30 sec. The next steps were then repeated 22 times: 95°C for 30 sec, 62°C for 30 sec, 72°C for 30 sec, ending with a single incubation at 72°C for 5 min and a hold at 4°C. Fifteen µl of the reaction mixture was removed and electrophoresed on a 1.5% agarose gel. The primer sequences used for RT-PCR were as follows: actin 5′-ACCTTCAACTCCATCATGAAG-3′ and 5′-CTGCTGGAAGGTGGACAG-3″, canine VAMP1 5′-ACAGCAAACCCAGGCACAAGTGG-3′ and 5′-TGGCACAGATAGCTCCCAGCAT-3′, canine VAMP2 5′-TGGAGCGGGACCAGAAGCTGT-3′ and 5′-TTTGCGCTTGAGCTTGGCTGC-3′, canine VAMP3 5′-CTGCCACCCCTAAGGATCAA-3′ and 5′-CCCACGCTGAATTTGAGAGG-3′, canine VAMP4 5′-ACCGCGCTTGATTTGGTGACA-3′ and 5′-GGCAAAACAGAGGCTGGTTATCTGC-3′, canine VAMP5 5′-GCTCCACATGCCCAGGACGC-3′ and 5′-TGCCCTCAAGGGCCAGTCTGT-3′, canine VAMP7 3′-AGTGGTGGAGACTCAAGCCCA-5′ and 5′-ATCCACCACAGAGAGGTGACACA-3′, canine VAMP8 5′-TTTCGCCACCCATGCCATCCC-3′ and 5′-ACCGGCCACCATCAGTGTCCT-3′, rat VAMP7 5′-GTTGCCAGGGGAACCACTAT-3′ and 5′-GCCAGAACGCTCGAAAACTC-3′, α-gal A 5′-TGTGCAACGTTGACTGCCAAGAAG-3′ and 5′-TCCTGCAGGTTTACCATAGCCACA-3′. For qPCR RNA was extracted and converted to cDNA as described above. SYBR® Select Master Mix (Applied biosystems) was added to triplicate samples of cDNA and primers. The primer sequences used for qPCR were as follows: actin 5′-GATCAAGATCATCGCACCCC-3′ and 5′-ACAGTCCGCCTAGAAGCATT-3′, VAMP1 5′-GGAGGAAGTGGTGGACATCAT-3′ and 5′-GCTGTGACAGTATCTCATCCCT-3′ VAMP2 5′-GGAGGATGGGTCGGCTAC-3′ and 5′-TCTCCTGTTACTGGTGAGGTT-3′, VAMP3 ATCGTGCAGACGCGCTA and CCTATCGCCCACATCTTGC, VAMP4 5′-GGTGGCGTGGATGCAAAATAA-3′ and 5′-GCGCGGTATTTCAAGACTGT-3′, VAMP5 5′-GCCTTCAGCAAGACAACCAA-3′ and 5′-TAGATCCGGCAACGGACATT-3′, VAMP7 5′-GGAGGATTTTGAACGTTCCCG-3′ and 5′-GATGCTTCAACTGTGCAGCC-3′, VAMP8 5′-GATCTGGAAGCCACATCGGA-3′ and 3′-GTGGCGAAAAGCACGATGAA-3′, α-gal A 5′-TCTTGGCCTGGACATCTTCT-3′ and 5′-TCACTAGCATATCTGGGTCGT-3′. SYBR-Green fluorescence was detected using a CFX96™ Real-Time System with thermal cycling controlled by a C1000 Touch™ Thermal Cycler (Bio-Rad) under standard cycling mode. Reactions were run using the following parameters: UDG activation: 2 min at 50°C; DNA polymerase activation: 2 min at 95°C, and 40 cycles of denaturing at 95°C for 15 s and annealing/extending at 60°C for 1 min. A dissociation curve was generated following each run and the derivative plot of melting temperatures was assessed for the presence of contaminating products. Five-point standard curves were generated for each set of primers using 10-fold dilutions of cDNA obtained from a pool of three control samples of MDCK cells. Real-time PCR efficiencies for each set of primers were determined according to the following equation: E = 10^(−1/slope)^. Primers that differed by less than 10% in efficiency were considered of similar efficiencies for calculation purposes. Relative gene expression for individual VAMPs upon VAMP7 or VAMP8 knockdown was calculated using the comparative Ct method (ΔΔCt) with actin as the reference gene. To assure precision among replicates, only triplicates that generated a standard deviation of less than 0.250 were considered in the analysis to allow the ability to discriminate between 2-fold dilutions in 95% of the cases.

### Cell Culture and Adenovirus Infection

Rat adrenal pheochromocytoma cells [PC-12 cells [69]; provided by Manojkumar Puthenveedu] were grown in DMEM with 5% FBS and 10% horse serum. MDCK cells were cultured in Minimum Essential Medium (Sigma) supplemented with 10% FBS. For adenoviral infection, MDCK T23 cells were cultured for four days on 12-mm Transwell permeable supports (0.4-µm pore; Costar, Cambridge, MA), rinsed extensively with PBS and incubated for 1 h at 37°C with 150 µl of PBS/virus on the apical surface of the Transwell and 1 mL PBS on the basolateral surface. These cells stably express the tetracycline transactivator which drives expression of many of our adenoviral constructs [70]. MDCK T23 cells were used for delivery assays and the parental cell line MDCK 2001 (used to generate MDCK T23 cells) was used for experiments measuring cilia length.

### DNA and Adenoviral Constructs

Generation of replication-defective recombinant adenoviruses encoding influenza HA, endolyn, and Ensol are described in [15], [71]–[73]. cDNA encoding GFP-VAMP7 was kindly provided by Sergio Grinstein. VAMP7-HA was generated from full length human VAMP7 pCMV Sport6 cDNA (Thermo Scientific clone ID 6503665). The open reading frame was amplified using PCR with 5′-CACCATGGCGATTCTTTTTGCTGTTGTTGC-3′ and 5′-CTAAGCGTAGTCTGGGACGTCGTATGGGTATTTCTTCACACAGCTTGG-3′ primers and inserted using the pcDNA3.1-TOPO kit (Invitrogen). Rab4-GFP, and Rab5-GFP, and Rab11a-GFP cDNAs were gifts from Jim Goldenring. Rab11a was SNAP tagged by PCR amplification of Rab11a-GFP and then subcloned into the BamHI and XhoI sites of pSNAP-tag(m) vector using the following primers: 3′-TAGGGATCCATGGGCACCCGCGACGACGA-3′ and 5′-CTAG CTCGAG CTAGATGTTCTGACAGCACT-3′.

### SiRNA Oligonucleotides and Transfection

MDCK cells were trypsinized (day 1) and plated to be ∼80% confluent the next day (∼3×10^6^ cells in a 10 cm dish). On day 2, transfection reagents were prepared while the cells were trypsinized. For each sample, 75 pmol of siRNA oligo (Sigma) was added to 62.5 µl OptiMEM (Invitrogen). Lipofectamine (Invitrogen; 3.75 µl in 62.5 µl OptiMEM) was added and the mixture was incubated for 20 min. The cells were collected and resuspended at a concentration of 2.4×10^6^ cells/mL. Cells (330 µl) were mixed with 125 µl of siRNA/Lipofectamine/OptiMEM and plated on each Transwell. Cells were used for experiments on day 6. PC-12 cells were transfected on day 1 using AMAXA electroporation; 2×10^6^ cells were mixed with 200 pmol siRNA and electroporated with program U-029, then plated onto coverslips in four wells of a 12-well dish. The next day, the cells were transfected again using RNAiMAX (Invitrogen) following the manufacturer’s protocol. On day 3 the cells were serum starved, and the following day they were processed for immunofluorescence or RT-PCR. The siRNA target sequences are as follows: control (firefly luciferase) 5′-GAAUAUUGUUGCACGAUUU-3′, VAMP8 canine 5′-CCACATCGGAGCACTTCAA-3′, VAMP7 canine 5′-GAAGAGGTTCCAGACTACA-3′, VAMP7 rat 5′-GAAGAGGTTCCAGACCACA-3′, VAMP7 #2 5′-GTGGAGGAAACTTCCTGGAG-3′, α-gal-A 5′-GATAGATCTGCTGAAATT-3′.

### Indirect Immunofluorescence

Cells were fixed in paraformaldehyde and processed for indirect immunofluorescence as described in [68]. Polyclonal anti-giantin antibody was a gift from Adam Lindstedt, EEA1 (BD; 1∶1000); Anti-LAMP2 AC17 monoclonal antibody was a gift from Enrique Rodriguez-Boulan; anti-HA epitope tag monoclonal antibody (Covance; 1∶500) was used for HA tagged proteins. Anti-septin 7 polyclonal antibody (1∶500) was a gift from Elias Spiliotis; polyclonal anti-syntaxin 3 (Abcam) was used at 1∶200; polyclonal anti-BBS3/Arl6 (Santa Cruz) was used at 1∶300; TMR-STAR (New England Biolabs; 3 µM final concentration) was used to label SNAP-tagged proteins. AlexaFlour conjugated secondary antibodies were from Invitrogen and used at 1∶500. Samples were mounted in ProLong Gold Antifade with DAPI (Invitrogen). Confocal images were acquired on a Leica SP5 confocal microscope (100x/1.5 NA objective) and processed using Adobe Photoshop. To determine Pearson’s Correlation Coefficients, confocal stacks were opened in Imaris and a median filter (3×3×1) was applied. Images were subjected to automatic thresholding using the method from [74]. A region of interest was selected for calculation of colocalization using the Imaris Coloc function. In order to determine lysosome volume, confocal stacks of LAMP2 stained cells were used to produce a 3D reconstruction of lysosomal volume using Imaris software using its Surfaces function, with automatic threshold setting. For VAMP7 and control siRNA treated cells, the average individual lysosome volume was obtained in each stack image and lysosome volume for VAMP7 knockdown cells was normalized against the average value obtained for cells treated with control siRNA. Two-sample t-test was used to assess statistical significance with α of 0.05. For VAMP7 overexpression experiments, sub-regions of fields where GFP-VAMP7 was expressed were selected, paired with identically sized areas in the same fields that were absent for GFP-VAMP7 expression, and average lysosome volume was obtained for each region. Lysosome volumes in GFP-VAMP7-expressing cells were normalized to the average volumes measured in non-expressing regions and statistical significance was assessed using paired t-test with α of 0.05.

### Biosynthetic Surface Delivery Kinetics

MDCK cells cultured on Transwells for four days were infected with replication-defective recombinant adenoviruses encoding HA at a multiplicity of infection (MOI) of 25, endolyn (MOI 100), or ensol (MOI 50) as described above. The following day, cells were rinsed with PBS^++^ (Sigma) and incubated in medium A (cysteine-free, methionine-free MEM with 0.35 g/liter NaHCO_3_, 10 mM MES, and 10 mM HEPES, pH 7.0) for 30 min in a 37°C water bath. The filters were incubated on a 50 µl drop of [^35^S]-Easy Tag (2.5 µl/well for HA) or ^35^S-cysteine (5 µl/well for endolyn and Ensol) for 30 min at 37°C. The cells were rinsed once with warm medium A. Warm media with cysteine and methionine was added to chase the cells for the indicated time points. HA was then trypsinized as described in [71] and endolyn was biotinylated as described in [72]. To quantitate Ensol secretion kinetics, apical and basolateral media were collected and replaced at each time point, and cells were solubilized at the final time point [15]. Ensol was immunoprecipitated from each sample using anti-endolyn antibody. Protein samples were separated by SDS-PAGE on 4–15% gradient gels (BioRad). Gels were dried and imaged on phosphoimager screens (BioRad).

### Measurement of Cilia Length

siRNA-transfected MDCK cells grown on Transwells and PC-12 cells cultured on coverslips were fixed and processed for indirect immunofluorescence as described above. Anti-acetylated tubulin monoclonal antibody (Sigma) was used at a dilution of 1∶400 to visualize cilia, with secondary antibody and mounting media as described above. Multiple fields of cells were acquired using a Leica DM6000B epifluorescence microscope with a 100x/1.4 NA objective. Images were opened in image J and individual cilia were traced using the freehand drawing tool to measure length, standardized by tracing the scale bar. Nuclei in each field were counted to calculate the percent of ciliated cells.

### Measurement of Cyst Formation

MDCK cells transfected with siRNA were plated on plastic for 1 day then trypsinized and 15,000 cells were resuspended in 80 µl basement membrane Matrigel (BD Biosiences). The Matrigel was allowed to solidify at 37°C in a 5% CO_2_ incubator for 30 minutes before addition of growth medium. The media was changed every other day for 6 days. Indirect immunofluorescence of cysts was performed as described in [44]. Knockdown was confirmed by RT-PCR in duplicate siRNA- treated samples grown on plastic.

## Supporting Information

Figure S1 **qPCR analysis of VAMP levels in siRNA treated cells.** Relative expression of VAMP1, VAMP2, VAMP3, VAMP4, VAMP5, VAMP7 and VAMP8 in cells transfected with VAMP7 or VAMP8 siRNA was quantified over 15 experiments, totaling 4 to 35 replicates for each condition. Actin was used as a reference gene and relative expression to samples treated with control siRNA was quantified using the ΔΔCt method. Median values +/−95% confidence intervals are plotted. *p<0.05 using confidence interval analysis for null of 1.0.
(TIF)

Figure S2 **VAMP7 knockdown decreases cilia frequency and cilia length in PC-12 cells.** PC-12 cells were transfected with siRNA on day 1 and transfected again the following day. On day 3 the cells were supplemented with serum free media, and 24 hours later were harvested for RT-PCR (A) or processed for indirect immunofluorescence to stain cilia. (B) The percent of cells with a primary cilium was quantitated as described in Methods and was significantly decreased in VAMP7-depleted cells (mean +/− SEM of three experiments is plotted; *p<0.05 assessed by Student’s t-test). (C) Cilia lengths from three experiments were plotted as in Figure 4. VAMP7 knockdown significantly reduced cilia length in three experiments (*p<0.05 determined by Mann-Whitney Rank Sum Test of median cilia length from three experiments). Control n = 586 cells, VAMP7 KD n = 468 cells.
(TIF)

Figure S3 **VAMP7 knockdown does not alter the localization of proteins required for ciliary biogenesis.** (A) Control and VAMP7 depleted MDCK cells were fixed and processed for indirect immunofluorescence to detect syntaxin 3 (Syn3) and acetylated tubulin (Ac Tub) and imaged using confocal microscopy. A maximal projection of apical sections that include the primary cilia and a single lateral section are shown. (B and C) Control and VAMP7 depleted MDCK cells were fixed and processed for indirect immunofluorescence to detect septin 7 (Sept 7) and acetylated tubulin. Panels in B show apical and lateral confocal sections of septin 7 distribution in cells, and panels in C show that colocalization of a subset of Septin 7 with acetylated tubulin persists upon VAMP7 knockdown. (D) The localization of Arl6/BBS3 to cilia and sub-ciliary structures was examined in control and VAMP7 depleted cells using confocal microscopy. Scale bars: 10 µm.
(TIF)
