# Supplementary figures and images for: VAMP7 Modulates Ciliary Biogenesis in Kidney Cells
Source: PLoS One. 2014 Jan 22;9(1):e86425. doi: 10.1371/journal.pone.0086425 (PMC3899255; doi:10.1371/journal.pone.0086425)

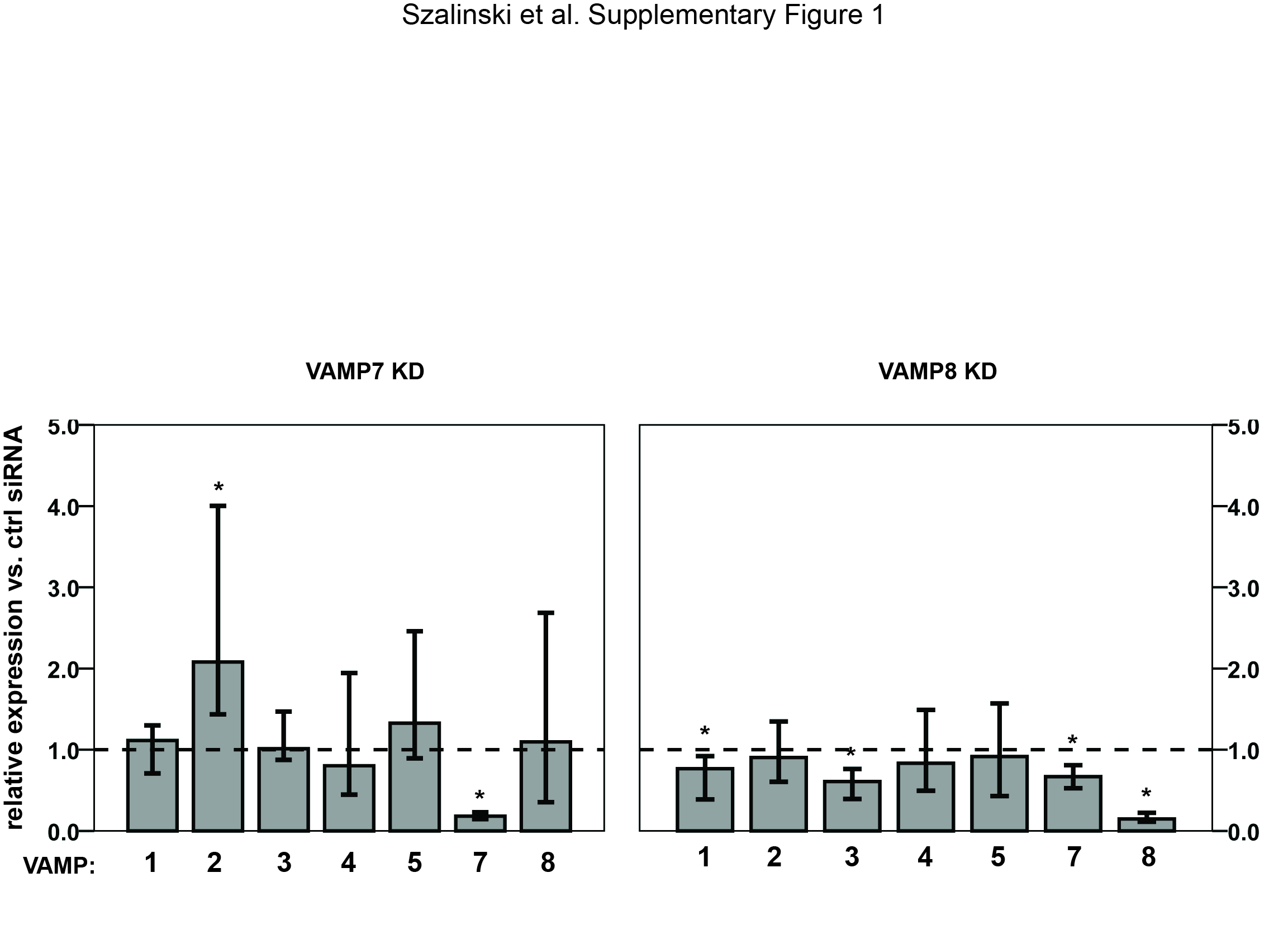

Supplement: Figure S1 — qPCR analysis of VAMP levels in siRNA treated cells. Relative expression of VAMP1, VAMP2, VAMP3, VAMP4, VAMP5, VAMP7 and VAMP8 in cells transfected with VAMP7 or VAMP8 siRNA was quantified over 15 experiments, totaling 4 to 35 replicates for each condition. Actin was used as a reference gene and relative expression to samples treated with control siRNA was quantified using the ΔΔCt method. Median values +/−95% confidence intervals are plotted. *p<0.05 using confidence interval analysis for null of 1.0. (TIF) [file pone.0086425.s001.tif]

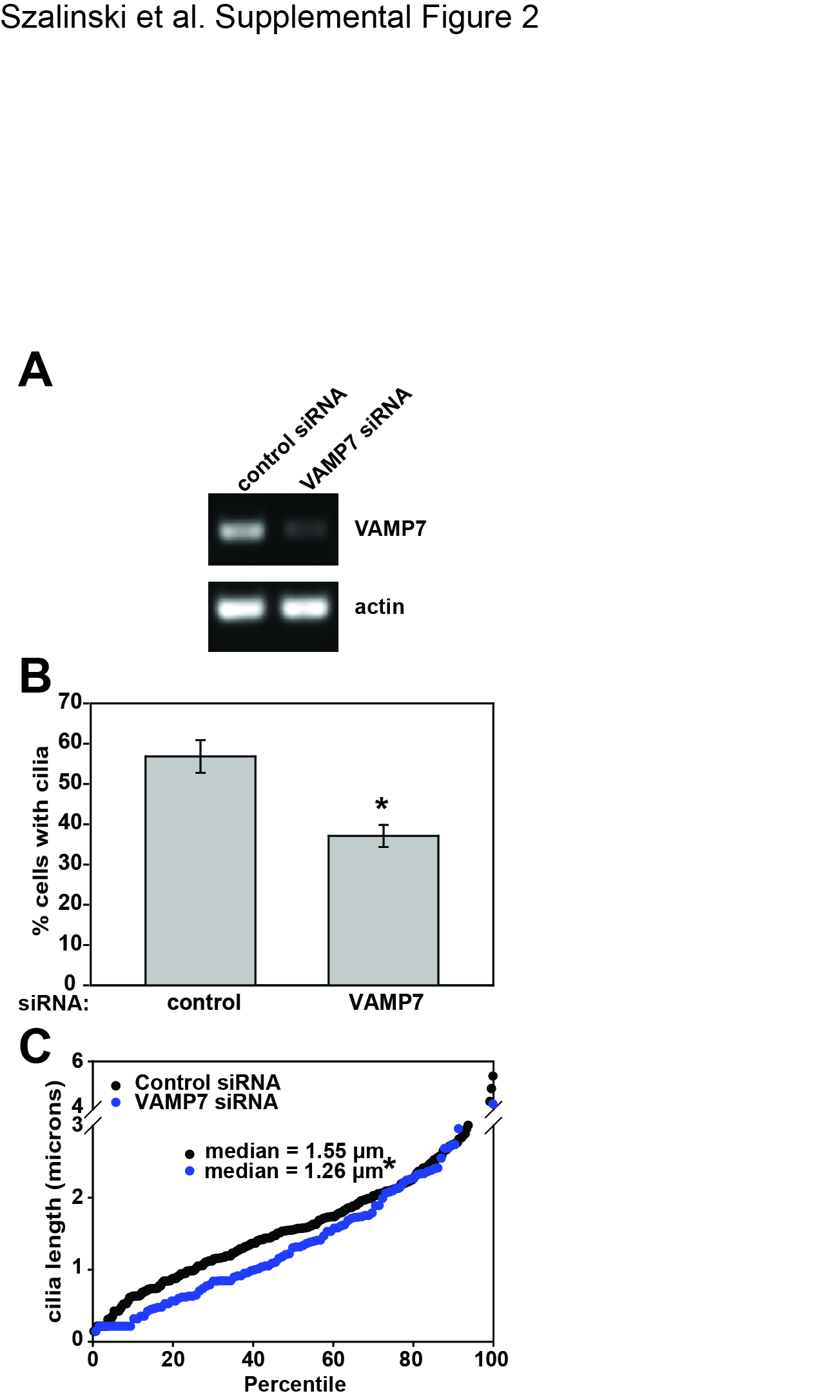

Supplement: Figure S2 — VAMP7 knockdown decreases cilia frequency and cilia length in PC-12 cells. PC-12 cells were transfected with siRNA on day 1 and transfected again the following day. On day 3 the cells were supplemented with serum free media, and 24 hours later were harvested for RT-PCR (A) or processed for indirect immunofluorescence to stain cilia. (B) The percent of cells with a primary cilium was quantitated as described in Methods and was significantly decreased in VAMP7-depleted cells (mean +/− SEM of three experiments is plotted; *p<0.05 assessed by Student’s t-test). (C) Cilia lengths from three experiments were plotted as in Figure 4. VAMP7 knockdown significantly reduced cilia length in three experiments (*p<0.05 determined by Mann-Whitney Rank Sum Test of median cilia length from three experiments). Control n = 586 cells, VAMP7 KD n = 468 cells. (TIF) [file pone.0086425.s002.tif]

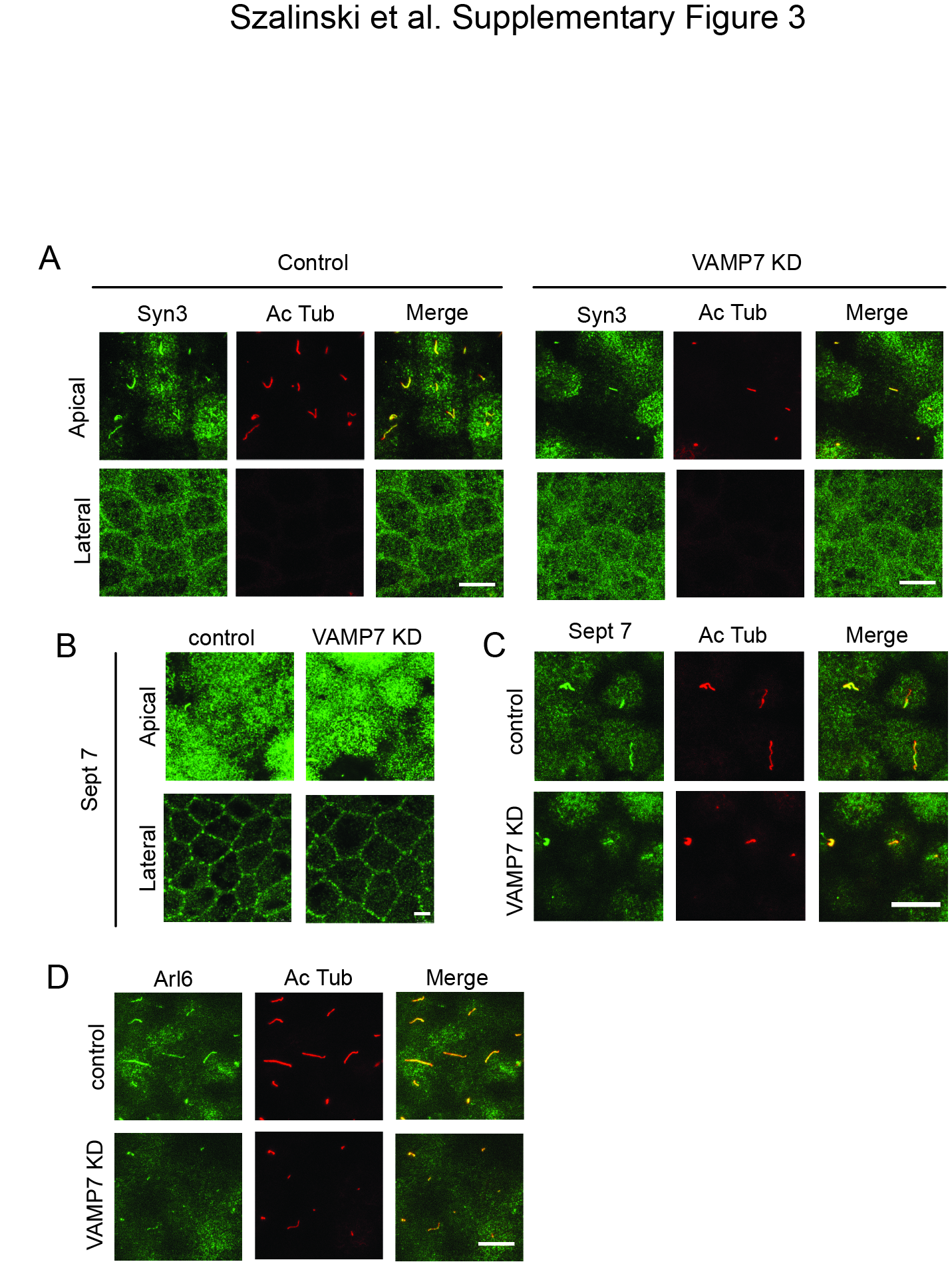

Supplement: Figure S3 — VAMP7 knockdown does not alter the localization of proteins required for ciliary biogenesis. (A) Control and VAMP7 depleted MDCK cells were fixed and processed for indirect immunofluorescence to detect syntaxin 3 (Syn3) and acetylated tubulin (Ac Tub) and imaged using confocal microscopy. A maximal projection of apical sections that include the primary cilia and a single lateral section are shown. (B and C) Control and VAMP7 depleted MDCK cells were fixed and processed for indirect immunofluorescence to detect septin 7 (Sept 7) and acetylated tubulin. Panels in B show apical and lateral confocal sections of septin 7 distribution in cells, and panels in C show that colocalization of a subset of Septin 7 with acetylated tubulin persists upon VAMP7 knockdown. (D) The localization of Arl6/BBS3 to cilia and sub-ciliary structures was examined in control and VAMP7 depleted cells using confocal microscopy. Scale bars: 10 µm. (TIF) [file pone.0086425.s003.tif]
